# Adjusting for multiple prognostic factors in the analysis of randomised trials

**DOI:** 10.1186/1471-2288-13-99

**Published:** 2013-07-31

**Authors:** Brennan C Kahan, Tim P Morris

**Affiliations:** 1 MRC Clinical Trials Unit, Aviation House, 125 Kingsway, London WC2B 6NH, UK; 2 Hub for Trials Methodology Research, MRC Clinical Trials Unit, Aviation House, 125 Kingsway, London WC2B 6NH, UK

**Keywords:** Randomised controlled trial, Stratified randomisation, Restricted randomisation, Covariate adjusted analysis, Stratified analysis

## Abstract

**Background:**

When multiple prognostic factors are adjusted for in the analysis of a randomised trial, it is unclear (1) whether it is necessary to account for each of the strata, formed by all combinations of the prognostic factors (stratified analysis), when randomisation has been balanced within each stratum (stratified randomisation), or whether adjusting for the main effects alone will suffice, and (2) the best method of adjustment in terms of type I error rate and power, irrespective of the randomisation method.

**Methods:**

We used simulation to (1) determine if a stratified analysis is necessary after stratified randomisation, and (2) to compare different methods of adjustment in terms of power and type I error rate. We considered the following methods of analysis: adjusting for covariates in a regression model, adjusting for each stratum using either fixed or random effects, and Mantel-Haenszel or a stratified Cox model depending on outcome.

**Results:**

Stratified analysis is required after stratified randomisation to maintain correct type I error rates when (a) there are strong interactions between prognostic factors, and (b) there are approximately equal number of patients in each stratum. However, simulations based on real trial data found that type I error rates were unaffected by the method of analysis (stratified vs unstratified), indicating these conditions were not met in real datasets. Comparison of different analysis methods found that with small sample sizes and a binary or time-to-event outcome, most analysis methods lead to either inflated type I error rates or a reduction in power; the lone exception was a stratified analysis using random effects for strata, which gave nominal type I error rates and adequate power.

**Conclusions:**

It is unlikely that a stratified analysis is necessary after stratified randomisation except in extreme scenarios. Therefore, the method of analysis (accounting for the strata, or adjusting only for the covariates) will not generally need to depend on the method of randomisation used. Most methods of analysis work well with large sample sizes, however treating strata as random effects should be the analysis method of choice with binary or time-to-event outcomes and a small sample size.

## Background

Some randomised controlled trials (RCTs) adjust their analyses for prognostic factors which are thought to influence outcome (such as age or disease stage). This is commonly done to increase power [1-7], to guard against chance imbalances between treatment arms [3,8], or because the prognostic factors have been used as balancing variables in the randomisation process and it is necessary to account for them in the analysis to obtain correct type I error rates [9-14]. There are often several available methods to account for covariates in a trial analysis. For example, with a binary outcome either logistic regression with the prognostic factors as covariates or a Mantel-Haenszel technique may be used to estimate the treatment effect. Alternatively, one could adjust for the individual strata, formed by all combinations of the covariates, using a logistic regression model with the strata modelled as either fixed or random effects.

Although many articles have highlighted the benefits of covariate adjustment [1-8], relatively little attention has been paid to the best method of adjustment with multiple prognostic factors (with only one prognostic factor most methods of adjustment will give a similar answer [3]). Likewise, little research has looked into whether the type of adjustment should match the type of randomisation (e.g. a stratified analysis for stratified randomisation). When randomisation is carried out within each stratum (for example using stratified permuted blocks [15]), not only will each stratification factor be balanced between treatment arms, but each combination of stratification factors (i.e. each stratum) will as well. If there is an interaction between balancing factors (e.g. if the effect of age on outcome depends on the patient’s disease stage), it may be necessary to account for not only the stratification factors, but also their interactions (or for each stratum) in the analysis in order to obtain correct type I error rates [9]. Conversely, when randomised is not carried out within strata (e.g. when balancing factors are not used in the randomisation process, or when covariates are balanced marginally), it may be unnecessary to account for interactions between covariates to obtain correct type I error rates (although adjustment for strong interactions may lead to increased power). Minimisation [15] is the most commonly used method of balancing covariates marginally [10]. Briefly, the balance between treatment groups is calculated for each covariate, and then summed across all covariates to give an overall measure of balance. The patient is then allocated to the group that would give the best overall balance (usually with an element of probability [16]). Thus, treatment assignments are balanced within a covariate considered individually, but not within combinations of balancing covariates. This implies that for minimisation, adjusting only for the covariates used in the minimisation process (and not for their interactions, or equivalently each strata) should give valid type I error rates.

The goals of this paper are (1) to determine whether a stratified analysis is necessary to maintain correct type I error rates after randomisation is performed within each stratum (stratified randomisation), and (2) to compare different methods of adjustment in terms of type I error rate and power, irrespective of the method of randomisation used. We do not consider the issue of how best to adjust for centre-effects in multicentre RCTs, as this issue has different considerations, and has been discussed previously [3,14,17-21]. We also do not consider the case of treatment-by-covariate (or treatment-by-strata) interaction (that is, all analysis methods presented here assume the treatment effect is constant across different covariates or strata).

## Methods

### Methods of adjustment

Methods of adjustment for covariates will generally fall into two classes. The first involves adjusting only for the individual covariates. We refer to this as a *covariate-adjusted analysis*. The second involves adjusting for each individual stratum. We refer to this as a *stratified analysis*. We illustrate these two different approaches using an example. Suppose we wish to account for two binary covariates in the analysis: gender (male vs. female) and disease stage (early vs. late). A covariate-adjusted analysis would involve adjusting for gender and disease stage as two separate variables in a regression model. A stratified analysis however would account for each stratum formed by these covariates (male/early stage vs male/late stage vs female/early stage vs female/late stage). This could be done by adjusting for each stratum in a regression model using three dummy variables, but is often done by performing the analysis within each stratum, and combining the results. Examples of this include Mantel-Haenzel for binary outcomes, or a stratified Cox model for time-to-event outcomes.

We illustrate some general approaches to adjustment using the previous example, where there were two covariates of interest, gender and disease stage. Let *X*_*G*_ be a binary variable indicating the patient’s gender (0 = female, 1 = male), *X*_*S*_ be a binary variable indicating the disease stage (0 = early, 1 = late), and *X*_*treat*_ be a binary variable indicating whether the patient received the treatment or not.

Then, a covariate-adjusted analysis can be carried out by adjusting for the individual covariates in a regression model. This can be done with a model of the form:

(1) $$f\left(Y\right)=\alpha +\beta_{\mathrm{treat}}X_{\mathrm{treat}}+\beta_{G}X_{G}+\beta_{S}X_{S}$$

Where *Y* is the patient outcome, *f(.)* is the link function, *α* the intercept, and the *β*’s represent regression coefficients.

There are several methods of performing a stratified analysis, some of which apply only to certain outcome types. One method of performing a stratified analysis is to account for all the strata in a regression model using indicator variables. This can be thought of as a stratified analysis using fixed effects (this is a common method of analysis for multicentre trials with continuous outcomes [14]). Since gender and disease stage form four strata (female/early, female/late, male/early, male/late) we need three dummy variables. Let *X*_*FL*_ be a binary variable indicating whether the patient was female with late-stage disease (0 = no, 1 = yes), *X*_*ME*_ be a binary variable indicating whether the patient was male with early-stage disease (0 = no, 1 = yes), and *X*_*ML*_ be a binary variable indicating whether the patient was male with late-stage disease (0 = no, 1 = yes) (the choice of which stratum to drop from the parameterisation is arbitrary, and will have no impact on the estimated treatment effect or its standard error).

Then, a stratified analysis using fixed effects can be performed using the following model:

(2) $$f\left(Y\right)=\alpha +\beta_{\mathrm{treat}}X_{\mathrm{treat}}+\beta_{\mathrm{FL}}X_{\mathrm{FL}}+\beta_{\mathrm{ME}}X_{\mathrm{ME}}+\beta_{\mathrm{ML}}X_{\mathrm{ML}}$$

It should be noted that (provided there are no continuous covariates) equation (2) is equivalent to adjusting for all the individual covariates as well as all interactions in the sense that the treatment effect and its standard error will be identical.

Another method of performing a stratified analysis is to treat strata as random effects from a distribution (which is also sometimes used to analyse multicentre trials [14]). This can be thought of as a stratified analysis using random effects. Consider the scenario where we have *j* strata (in the above example we have four strata). A stratified analysis using random effects could then be performed using the following model:

(3) $$f\left(Y\right)=\alpha +u_{j}+\beta_{\mathrm{treat}}X_{\mathrm{treat}}$$

where *u*_*j*_ is a random effect for the *j*th stratum. *u*_*j*_ would generally be assumed to follow a normal distribution. This assumption is likely to be violated in many scenarios, but previous research has shown that the fixed parameters from random effects models are robust to misspecification of the random effects distribution [14,22].

As mentioned previously, some types of stratified analyses can only be performed for specific outcome types. Two examples of this are a Mantel-Haenszel analysis and a stratified Cox model. Mantel-Haenszel applies to binary outcomes, and involves calculating an odds ratio within each stratum, then calculating a weighted average of the results to get a final estimate. A stratified Cox model is performed with time-to-event outcomes, and involves calculating a hazard ratio within each stratum, then combining the results for a final estimate.

For continuous outcomes, the interpretation of the treatment effect will not depend on the method of analysis. However, for binary or time-to-event outcomes, adjustment for different factors can lead to different estimates [23,24], and therefore must be interpreted based on the adjustment factors. If the interactions between prognostic factors are large, a stratified-analysis may lead to larger estimates of treatment effect compared with a covariate-adjusted analysis, and may therefore increase power [23,24].

### Simulation study based on theoretical data

We performed a simulation study to determine (i) if a stratified analysis is necessary after stratified randomisation to maintain correct type I error rates, and (ii) whether a covariate-adjusted analysis is adequate after minimisation for correct type I error rates (rendering a stratified analysis unnecessary).

We used two different methods of randomisation; (i) stratified permuted blocks, with a block size of 2, and (ii) minimisation with a random component of 80%. For each method of randomisation we performed two analyses; (a) covariate-adjusted analysis, using equation 1, and (b) stratified analysis using fixed effects, using equation 2 (which, as noted previously, is the same as model (4), and so is equivalent to the data generating model). We therefore assessed four randomisation-analysis combinations in total:

a) Stratified permuted blocks, with a covariate-adjusted analysis

b) Stratified permuted blocks, with a stratified analysis

c) Minimisation, with a covariate-adjusted analysis

d) Minimisation, with a stratified analysis

Our hypothesis was that using a covariate-adjusted analysis after stratified randomisation would lead to inflated type I error rates when there were substantial interactions between prognostic factors, but that a stratified analysis would lead to nominal type I error rates. Conversely, we hypothesised that a covariate-adjusted and a stratified analysis would lead to nominal type I error rates after minimisation.

We generated continuous outcomes from the following model (which in this scenario is equivalent to model (2) above):

(4) $$Y_{i}=\alpha +\beta_{\mathrm{treat}}X_{\mathrm{treat}}+\beta_{1}X_{1}+\beta_{2}X_{2}+\beta_{12}X_{12}+\epsilon_{i}$$

where *Y*_*i*_ is the outcome from the *i*th patient, *X*_*1*_ and *X*_*2*_ are balancing factors and *X*_*12*_ is their interaction, *β*_*1*_ and *β*_*2*_ are the regression coefficients for those balancing factors, and *β*_*12*_ is the regression coefficient for their interaction. *ϵ*_*i*_ is a random error term, and is normally distributed with mean 0 and variance *σ*^*2*^.

We performed two sets of simulations. In the first, we varied the size of *β*_*12*_ while holding the other parameters constant. In the second, we varied the proportion of patients with *X*_*12*_ = 1 while holding the other parameters constant. More information on both of these scenarios is available below. We set the sample size to 250 patients, and used 5000 replications for each scenario to give a standard error of about 0.3% when estimating the type I error rate, assuming a true type I error rate of 5%.

#### Varying the size of the interaction term

For the first set of simulations we varied the size of *β*_*12*_ while holding the other parameters constant. We varied *β*_*12*_ from 0 to 3 in increments of 0.2. *β*_*1*_ and *β*_*2*_ were set to 0.5, *β*_*treat*_ was set to 0 and *σ*^*2*^ to 1. We set P(*X*_*1*_ = 1) = P(*X*_*2*_ = 1) = 0.5, and generated *X*_*1*_ and *X*_*2*_ independently. It follows that P(*X*_*12*_ = 1) = 0.25.

#### Varying the distribution of patients across strata

For the second set of simulations we varied the proportion of patients with *X*_*12*_ = 1 while holding the other parameters constant. We did this by varying P(*X*_*1*_ = 1) and P(*X*_*2*_ = 1) together from 0.1 to 0.5 in increments of 0.05. This corresponds to varying P(*X*_*12*_ = 1) from 0.01 to 0.25. All other parameters were set to the same values as above, except *β*_*12*_ which was set to 1.5 (which is 50% larger than *σ*, and is unlikely to occur often in practice, but is used here for the purposes of illustration).

### Simulation study based on real trial data

#### Methods

We performed a simulation study based on real datasets to (1) determine whether stratified randomisation is necessary to maintain correct type I error rates after stratified randomisation in real trial scenarios, and (2) to compare different methods of adjustment in terms of type I error rate and power, irrespective of the method of randomisation used. We used three datasets (one each with a continuous, binary, and time-to-event outcome), which are further described below.

When generating data, we used the linear predictor:

(5) $$\eta_{i}=\alpha +\beta_{\mathrm{treat}}X_{\mathrm{treat}}+\sum_{c=1}^{C}\beta_{c}X_{c}+\sum_{c=1}^{C}\sum_{d>c}\beta_{\mathrm{cd}}X_{\mathrm{cd}}$$

where *β*_*c*_ denotes the main effect from the *c*th covariate, and *β*_*cd*_ denotes the two-way interaction between the *c*th and *d*th covariates (for c ≠ d). More information on the exact data generating models can be found below.

For simplicity, we chose to simulate data based only on the two-way interactions between covariates, rather than including any three-way or higher interactions. It should be noted that although we simulated data based only on the two-way interactions, stratified analyses were performed adjusting for *all* interactions (or, equivalently, all strata), rather than only the two-way interactions.

We generated prognostic variables from a multivariate normal distribution with a covariance matrix based on the original data set so that the proportion of patients in each stratum was similar to the original study. We then categorised binary covariates using a cut-point specified to give the desired proportions in each group.

We randomised patients to one of two treatments using three different methods: (1) simple randomisation, where all patients had a 50% chance of either treatment; (2) stratified permuted blocks, with a block size of 4; and (3) minimisation, with a random element of 80% (i.e. patients were assigned to the preferred treatment arm with a probability of 80%).

As above, we used 5000 replications for each scenario. We compared different analysis methods in terms of the type I error rate and power. For continuous, binary, and time-to-event outcomes, the treatment effect was calculated as a difference in means, an odds ratio, and a hazard ratio respectively. To assess the type I error rate, we set *β*_treat_ to 0. To assess power, we set *β*_treat_ to give 80% power based on the specified sample size (for binary and time-to-event outcomes, we powered the study based on reducing, rather than increasing, the number of events).

##### MIST2 (continuous outcome)

For continuous outcomes, we based our simulations on the MIST2 trial, which has been described previously [9,10,25,26]. Briefly, MIST2 was a randomised controlled trial assessing whether tissue plasminogen activator, deoxyribonoclease, or their combination was effective in reducing the size of patients’ pleural effusion (a continuous outcome). Two hundred and ten patients were randomised using minimisation, with a random component of 80%. Balancing variables were the size of the baseline pleural effusion (greater or less than 30% of the hemithorax), whether the patient was purulent, and whether the infection was community or hospital acquired.

We generated data from the following model:

$$Y_{i}=\eta_{i}+\epsilon_{i}$$

where *Y*_*i*_ is the outcome for the *i*th patient, *η*_*i*_ is the linear predictor (as in equation 5), and *ϵ*_*i*_ is a random error term. The parameters for the covariates and their two-way interactions can be found in Table 1. *ϵ*_*i*_ was generated from a normal distribution with mean 0 and standard deviation 19.1.

**Table 1:** Parameters from the MIST2 dataset

**Variable**	**Proportion of patients with covariate**	**Regression parameter**
**Main effects**		
**Pleural effusion >30**%	67	−25.4
**Purulence**	49	1.8
**Hospital infection**	13	−7.0
**Interaction**		
**Pleural effusion X purulence**	29	3.5
**Pleural effusion X hospital infection**	7	24.4
**Purulence X hospital infection**	6	−1.6

We used sample sizes of 100, 200, 500, and 1000 patients. We used three methods of analysis; (a) covariate-adjusted analysis (equation 1); (b) stratified analysis using fixed effects (equation 2); and (c) stratified analysis using random effects (equation 3).

##### AUGIB (binary outcome)

For binary outcomes, we based our simulations on the Acute Upper Gastrointestinal Bleeding (AUGIB) audit dataset. This dataset has been described previously [27-30]. Briefly, this was an observational dataset collected on consecutive patients presenting with AUGIB in the UK. We used further bleeding as an outcome. We chose four prognostic factors; urea (as a binary covariate, dichotomised at its median), presence of shock, prolonged coagulation, and outpatient at admission. The dataset comprised of 4342 patients with complete data on the above covariates.

We generated latent outcomes using the following model:

$$Y_{i}^{*}=\eta_{i}+\epsilon_{i}$$

where *Y*_i_^*^ is a latent outcome for *i*th patient, *η*_*i*_ is the linear predictor (as in equation 5), and *ϵ*_*i*_ is a random error term that follows a logistic distribution with mean 0 and variance π^2^/3. Binary responses were generated as *Y*_*i*_ = 1 if *Y*_i_^*^ > 0, and 0 otherwise. The regression parameters for the covariates and their two-way interactions can be found in Table 2.

**Table 2:** Parameters from the AUGIB dataset

**Variable**	**Proportion of patients with covariate**	**Odds ratio**
**Main effects**		
**Outpatient**	83	0.32
**Shock**	36	2.83
**Urea > 9.1**	55	1.88
**Coagulation**	13	1.87
**Interaction**		
**Outpatient X shock**	30	1.07
**Outpatient X urea**	44	1.67
**Outpatient X coagulation**	10	1.97
**Shock X urea**	24	0.71
**Shock X coagulation**	6	0.79
**Urea X coagulation**	9	0.77

We used sample sizes of 100, 200, 500, 1000, and 2000 patients. We used four methods of analysis; (a) covariate-adjusted analysis (equation 1); (b) stratified analysis using fixed effects (equation 2); (c) stratified analysis using random effects (equation 3); and (d) Mantel-Haenszel estimates (a type of stratified analysis).

The event rate was approximately 23.6% in the control arm for all scenarios, except for a sample size of 100 where we used an event rate of about 30.1% in order to ensure an adequate number of events occurred in the treatment arm when assessing power.

##### PBC (time-to-event outcome)

For time-to-event outcomes, we based our simulations on the PBC trial. This dataset has been described previously [9,31]. Briefly, this was a randomised trial assessing whether D-penicillamine could increase overall survival time (primary outcome) in patients with primary biliary cirrhosis. We chose four prognostic factors; age, log(bilirubin), albumin (all as binary covariates, dichotomised at their medians), and disease stage (1/2 vs 3/4). The dataset comprised of 312 patients.

We generated time to event outcomes using the method described by Bender *et al.*[32]:

$$Y_{i}=H_{0}^{-1}\left[-\ln\left(U\right)\exp\left(-\eta_{i}\right)\right]$$

where *Y*_*i*_ is the time to death, *η*_*i*_ is the linear predictor (as in equation 5), *H*_*0*_ is the cumulative baseline hazard function, and *U* ~ Uniform (0, 1). This model implies proportional hazards. We censored event times at a cut-point specified to give a similar proportion of censoring as seen in the original dataset. The event rate was approximately 40.4% in the control arm for all scenarios. The regression parameters for the covariates and their two-way interactions can be found in Table 3.

**Table 3:** Parameters from the PBC dataset

**Variable**	**Proportion of patients with covariate**	**Hazard ratio**
**Main effects**		
**Age > 50 years**	51	3.01
**Log(bilirubin) > 0.3**	50	10.65
**Albumin > 3.55**	50	0.58
**Disease stage > 3**	73	5.02
**Interaction**		
**Age X log(bilirubin)**	24	1.03
**Age X albumin**	23	1.40
**Age X disease stage**	38	0.66
**Log(bilirubin) X albumin**	18	0.77
**Log(bilirubin) X disease stage**	42	0.45
**Albumin X disease stage**	32	0.65

We used sample sizes of 100, 200, 500, 1000, and 2000 patients. We used four methods of analysis; (a) covariate-adjusted analysis (equation 1); (b) stratified analysis using fixed effects (equation 2); (c) stratified analysis using random effects (equation 3); and (d) a stratified Cox model (a type of stratified analysis).

##### Sensitivity analysis

We performed a sensitivity analysis to assess whether increasing the size of the observed interactions in the MIST2, PBC, and AUGIB datasets had any impact on type I error rates. Simulations were performed as above, but we systematically increased the size of each interaction term by of a factor of 2.5, 5, 7.5, and 10. For example, the size of the observed interaction terms in the MIST2 dataset (Table 1) was −1.6, 3.5, and 24.4. Increasing these interactions by a factor of 2.5 for this sensitivity analysis led to interaction sizes of −4.0, 8.8, and 61.0 respectively.

## Results

### Simulation study based on theoretical data

#### Varying the size of the interaction term

**Figure 1:**
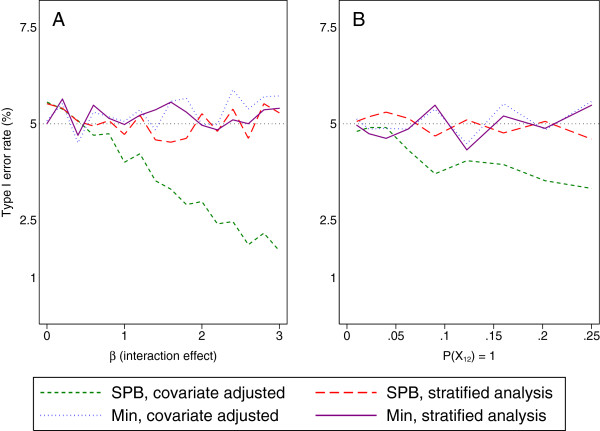
**Type I error rate with different randomisation and analysis methods. (A)** shows the type I error rate for different values of the interaction term between the two balancing factors. **(B)** shows the type I error rate for different values of P(X_12_ = 1) (which indicates the proportion of patients in the interaction group i.e. with both prognostic factors present). Two methods of randomisation were used (Min = minimisation, SPB = stratified permuted blocks), and two methods of analysis were used (covariate adjusted indicates the two balancing factors were used as covariates in a regression model; stratified analysis denotes that the strata formed from the combinations of the two balancing factors were entered as covariates in a regression model).

Results are shown in Figure 1a. When patients were randomised using minimisation, both a covariate adjusted analysis and a stratified analysis gave valid type I error rates, regardless of the size of the interaction. Likewise, when a stratified analysis was used after patients were randomised using stratified permuted blocks, error rates were nominal. However, a covariate adjusted analysis gave incorrect type I error rates whenever the interaction was ≠ 0.

This demonstrates that, in principle, when randomisation has been balanced within strata, a stratified analysis may be necessary to maintain nominal type I error rates when there are large interactions between balancing factors. For minimisation, which does not balance within strata, either a stratified or a covariate adjusted analysis will give valid type I error rates in the presence of large interactions.

#### Varying the distribution of balancing factors

Results are shown in Figure 1b. The impact of a covariate adjusted analysis after randomisation using stratified blocks on the type I error rate depended on the proportion of patients with *X*_*12*_ = 1; when this was small, type I error rates were close to nominal. However, as this increased, the type I error rates became too low.

This demonstrates that it is not only the size of the interaction which could impact the type I error rate under a covariate adjusted analysis, but also the distribution of patients across the different strata.

### Simulation study based on real trial data

#### MIST2 (continuous outcome)

**Figure 2:**
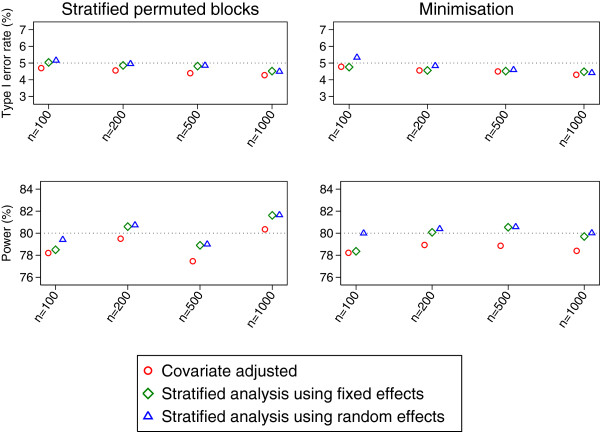
Type I error rate and power results for the MIST2 trial (continuous outcome).

Results after stratified permuted blocks and minimisation are shown in Figure 2. As expected, all methods of analysis (covariate-adjusted analysis, stratified analysis using fixed effects, and a stratified analysis using random effects) gave close to nominal type I error rates after simple randomisation (results not shown) or minimisation. Stratified analyses (using either fixed or random effects) gave valid results after randomisation using stratified permuted blocks. However, a covariate adjusted analysis also gave close to nominal type I error rates after stratified permuted blocks, contradicting results seen earlier.

A stratified analysis using random effects gave a small increase in power compared to either a covariate adjusted analysis or a stratified analysis using fixed effects with a sample size of 100 (approximately 1-2% across different randomisation methods). For larger sample sizes (between 200 and 1000 patients), stratified analyses using either fixed or random effects had similar levels of power, and were slightly more powerful than covariate adjusted analysis (approximately 1.5%).

Each method of analysis had a convergence rate of 100% in all scenarios.

#### AUGIB (binary outcome)

**Figure 3:**
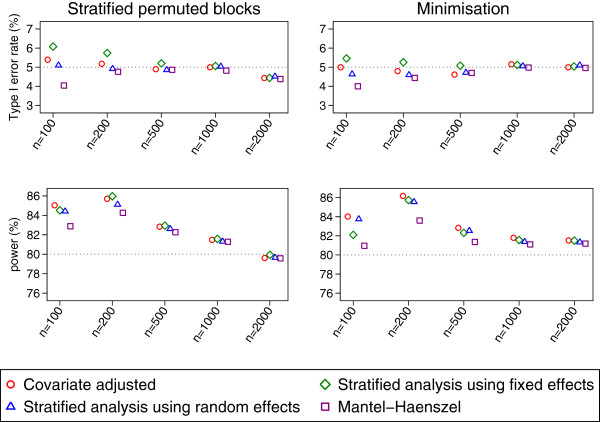
Type I error rate and power results for the AUGIB dataset (binary outcome).

Results after stratified permuted blocks and minimisation are shown in Figure 3. For larger sample sizes (500 or more patients), all analysis methods gave similar type I error rates and power; as above, a covariate-adjusted analysis gave correct type I error rates, even when used after stratified permuted blocks. Convergence rates were greater than 99% for all analysis methods.

For smaller sample sizes (100 or 200 patients), results for the different analysis methods were less similar. With 100 patients, all analysis methods apart from a stratified analysis using random effects had convergence issues; convergence rates for a covariate-adjusted analysis, a stratified analysis using fixed effects, and Mantel-Haenszel varied between 96-97%, whereas rates for a stratified analysis using random effects were >99%. All convergence rates were >99% with 200 patients.

The type I error rate for Mantel-Haenszel was too low with a sample size of 100 patients; this lead to a small loss in power (between 2-5% compared with a stratified analysis using random effects). With 200 patients, Mantel-Haenszel experienced a loss in power of 1-5% compared with a stratified analysis using random effects. Type I error rates and power results between a covariate-adjusted analysis and stratified analyses using either fixed or random effects were similar for 100 or 200 patients.

#### PBC (time-to-event outcome)

**Figure 4:**
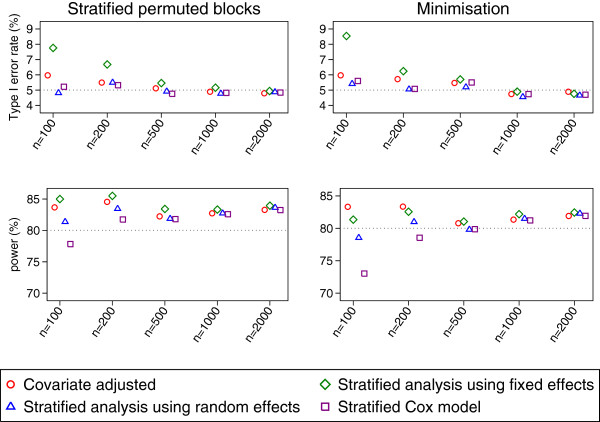
Type I error rate and power results for the PBC trial (time-to-event outcome).

Results after stratified permuted blocks and minimisation are shown in Figure 4. Convergence rates were above 99.9% for each method of analysis in all scenarios. For large sample sizes (1000 or 2000 patients) each method of analysis gave similar results; as above, a covariate-adjusted analysis gave correct type I error rates when used with stratified permuted blocks.

For other sample sizes (100, 200, and 500 patients) a stratified analysis using fixed effects had type I error rates that were too large (range across three different randomisation methods 7.4-8.5%, 6.2-6.7%, and 5.5-5.8% for 100, 200, and 500 patients respectively). Type I error rates were slightly too large for covariate-adjusted analyses, although less so than for stratified analyses using fixed effects (range across different randomisation methods 5.4-6.0%, 5.5-5.7%, and 5.1-5.7% for 100, 200, and 500 patients respectively). Conversely, stratified analyses using random effects and stratified Cox models gave nominal type I error rates (range across randomisation methods and sample sizes 4.7-5.5% and 4.4-5.6% for 100–500 patients for stratified analyses using random effects and stratified Cox models respectively).

Stratified analyses using fixed effects and covariate-adjusted analyses had highest power for smaller sample sizes, although this is likely a result of the inflated type I error rate associated with these analysis methods. Of the two analysis methods that gave nominal type I error rates for smaller sample sizes, stratified analyses using random effects had higher power than stratified Cox models (power increases of 3.6-5.5% and 1.7-2.5% for sample sizes of 100 and 200 respectively).

#### Sensitivity analysis

**Figure 5:**
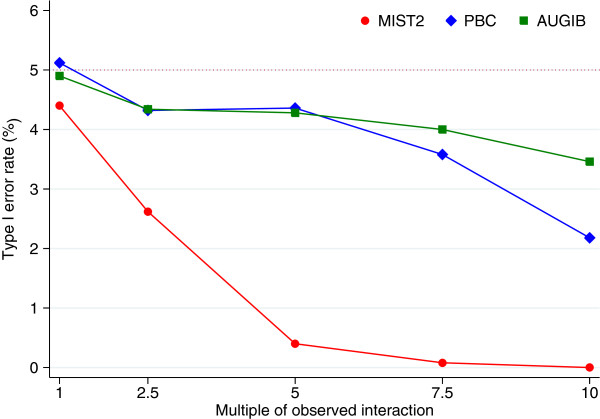
**Sensitivity analysis for MIST2, PBC, and AUGIB datasets.** Shows the type I error rate for the MIST2, PBC, and AUGIB datasets, after systematically increasing the size of the observed interactions.

Results are shown in Figure 5. For MIST2, the type I error rate was too low when the size of the observed interactions was increased by a factor of at least 2.5. For the PBC and AUGIB datasets, type I error rates were not substantially affected until the interactions had been increased by a factor of at least 7.5.

To be these results in perspective, increasing the observed interactions from the MIST2 dataset by a factor of 2.5 resulted in the effect size of the largest interaction being increased to almost 60 (approximately 3 times larger than the residual standard deviation). Increasing the observed interactions in the PBC and AUGIB datasets by a factor of 7.5 resulted in the odds ratio or hazard ratio of the largest interactions being increased to almost 399 and 162 respectively.

## Discussion

Our aims for this paper were to (1) determine whether it is necessary to perform a stratified analysis after using a randomisation method that balances within strata (such as stratified permuted blocks) to obtain correct type I error rates, and (2) to compare different methods of accounting for multiple prognostic factors in terms of power and type I error rates, irrespective of the method of randomisation.

Regarding point (1), it has previously been noted that for randomisation methods that balance within each stratum (e.g. stratified permuted blocks), it may be necessary to use a stratified analysis to obtain correct type I error rates when there are large interactions between balancing factors [9]. By comparison, this issue should not affect randomisation methods that do not balance within strata (e.g. simple randomisation, permuted blocks without stratification, or minimisation), and so both covariate-adjusted and stratified analyses should give correct results. We explored this issue using simulation under specific (and potentially unrealistic) conditions, and found that the necessity of a stratified analysis after stratified randomisation depended on (a) the size of the interactions between prognostic factors, and (b) the distribution of patients across strata. When there were both large interactions, and a relatively equal number of patients in each stratum, a covariate-adjusted analysis led to type I error rates that were too low. A stratified analysis by comparison gave valid results. However, when either the interactions were small, or there was a low percentage of patients in some strata, a covariate-adjusted analysis gave close to nominal type I error rates. As expected, both analysis methods gave valid results after minimisation. This is because minimisation balances baseline variables marginally, meaning that although variables are balanced, their interactions are not. Adjustment for the main effects will then be sufficient to obtain the nominal type I error rates.

In order to determine whether this issue was likely to affect real RCTs, we performed further simulations based on real data. Contrary to expectations, we found that a stratified analysis was not necessary after stratified randomisation; covariate-adjusted analyses lead to valid results in each of the three datasets we used. The reasons for this are not entirely clear. One possible explanation is that the interaction sizes we used (based on observed data) were not large enough to affect results. However, some of the interactions we used were substantial. For example, in the MIST2 trial one of the interactions was 28% larger than the standard deviation, in the AUGIB dataset one interaction had an odds ratio of 2.01 and another an odds ratio of 1.65, and in the PBC dataset one interaction had a hazard ratio of 0.45 and two others a hazard ratio of 0.67. Another explanation is that the distribution of patients across strata affected the results (i.e. some strata had too low a proportion of patients). This indicates that in practice, both large interactions between balancing factors and similar numbers of patients in most strata are necessary for a covariate-adjusted analysis to affect type I error rates after balancing within strata. However, the second condition may be unlikely; this would require a similar proportion of patients in each group for all balancing factors (i.e. close to 50% of patients in each level of a binary factor), as well as small correlations between balancing factors (as moderate to large correlations would lead to patients being much more likely to fall into certain stratum). In our view, these conditions seem unlikely to be met in practice. Given the imbalance in the number of patients in each stratum observed in the datasets, the size of the interactions would have to have been 2.5-7.5 times larger than they were in order to affect results. For the MIST2 trial for example, this would have required an interaction term of almost 60 (about three times larger than the standard deviation), which is not realistic in practice. We conclude that choosing between covariate-adjusted and stratified analyses does not need to be based on whether stratified randomisation was used.

This brings us to our second question; of the numerous methods of analysis available, which is most powerful, irrespective of the randomisation method used? For continuous outcomes, stratified analyses (either fixed or random effects) gave slightly higher power than a covariate-adjusted analysis, while all methods of analysis gave nominal type I error rates.

For binary and time-to-event outcomes, all methods of analysis gave similar results with large sample sizes. However, there were differences between analysis methods for small sample sizes. A stratified analysis using fixed effects led to inflated type I error rates in several scenarios, and cannot be recommended. A covariate-adjusted analysis also led to type I error rates that were too large when used with a time-to-event outcome; this is similar to results seen previously [9] where accounting for several balancing factors led to inflated type I error rates with a binary or time-to-event outcome.

Both Mantel-Haenszel for binary outcomes and a stratified Cox model for time-to-event outcomes gave close to nominal type I error rate (though Mantel-Haenszel was slightly too low in certain scenarios), but both suffered from a lack of power compared with other methods.

The one method of analysis which gave good results across all scenarios and sample sizes was a stratified analysis using random effects. While other analysis methods gave inflated type I error rates (stratified analysis using fixed effects, covariate-adjusted analysis) or led to a loss of power (Mantel-Haenszel, stratified Cox model) with a binary or time-to-event outcome and a small sample size, a stratified analysis using random effects gave nominal type I error rates and good power.

In this paper we have only considered methods of adjustment for prognostic covariates, and have not discussed ways to account for centre effects in multicentre RCTs. Comparison of methods for adjusting for centre effects in multicentre RCTs have been published previously [14,17,18]. However, many trials may adjust for both prognostic covariates and centre effects, and it is therefore worth considering whether the methods we have discussed in this paper will apply when also accounting for centre effects. A stratified analysis could be performed by accounting for the strata made up of all the centre-prognostic covariate combinations. However, if the number of centres is large, this may lead to over-stratification, which could lead to a loss of power. Therefore, we do not recommend this approach in general. An alternative approach is to perform a covariate-adjusted analysis for the prognostic factors (provided the sample size is large enough), and to account for centre-effects separately (e.g. using fixed or random effects, or generalised estimating equations). Alternatively, if centre effects are accounted for using fixed effects, a stratified analysis using random effects could be used for the prognostic factors.

One limitation of this paper is that we have dichotomised the continuous covariates from the original datasets for use in our simulation study. This has been done because a stratified analysis is only possible with categorical covariates. However, we would generally not recommend categorising continuous variables in practice. Provided the sample size is large enough, we would recommend accounting for continuous variables as covariates in a regression model. If there is a mixture of continuous and categorical variables, we could either perform a covariate-adjusted analysis for all variables, or perform a mixture of a covariate-adjusted and a stratified analysis, where the categorical covariates are grouped into strata and accounted for using random effects, and the continuous variables are included as covariates in the regression model.

## Conclusion

It is unlikely that a stratified analysis is necessary after stratified randomisation except in extreme scenarios. Therefore, the method of analysis (accounting for the strata, or adjusting only for the covariates) will not generally need to depend on the method of randomisation used. All of the methods of analysis considered in this article are acceptable with a continuous outcome, although when there are large interactions between covariates, a stratified analysis may increase power. With a binary or time-to-event outcome and a small sample size, we recommend the use of a stratified analysis using random effects, as this has been shown to maintain nominal type I error rates while giving high power. For binary or time-to-event outcomes with a large sample size, all methods of analysis are acceptable; however, it is often unclear what constitutes a large sample size. Therefore, if in doubt, we recommend the use of a stratified analysis using random effects to ensure correct type I error rates and power.

## Abbreviations

AUGIB: Acute upper gastrointestinal bleeding; MIST2: The second multicentre intrapleural sepsis trial; PBC: Primary biliary cirrhosis; RCT: Randomised controlled trial.

## Competing interests

Both authors declare that they have no competing interests.

## Authors’ contributions

BK devised the study, performed the simulations, and wrote the first draft of the manuscript. TM input into the manuscript. Both authors read and approved the final manuscript.

## Pre-publication history

The pre-publication history for this paper can be accessed here:

http://www.biomedcentral.com/1471-2288/13/99/prepub
